# Efficacy of Ciprofloxacin and Amoxicillin Removal and the Effect on the Biochemical Composition of *Chlorella vulgaris*

**DOI:** 10.3390/bioengineering9040134

**Published:** 2022-03-24

**Authors:** Rajamanickam Ricky, Fulvia Chiampo, Subramaniam Shanthakumar

**Affiliations:** 1 Department of Environmental and Water Resources Engineering, School of Civil Engineering, Vellore Institute of Technology (VIT), Vellore 632014, India; rickyraaaj@gmail.com (R.R.); shanthakumar.s@vit.ac.in (S.S.); 2 Department of Applied Science and Technology, Politecnico di Torino, Corso Duca degli Abruzzi 24, 10129 Torino, Italy

**Keywords:** ciprofloxacin, amoxicillin, algae, *Chlorella vulgaris*, biomass, removal

## Abstract

Antibiotics are frequently detected in the aquatic environment due to their excessive usage and low-efficiency removal in wastewater treatment plants. This can provide the origin to the development of antibiotic-resistant genes in the microbial community, with considerable ecotoxicity to the environment. Among the antibiotics, the occurrence of ciprofloxacin (CIP) and amoxicillin (AMX) has been detected in various water matrices at different concentrations around the Earth. They are designated as emerging contaminants (ECs). Microalga *Chlorella vulgaris* (*C. vulgaris*) has been extensively employed in phycoremediation studies for its acclimatization property, non-target organisms for antibiotics, and the production of value-added bioproducts utilizing the nutrients from the wastewater. In this study, *C. vulgaris* medium was spiked with 5 mg/L of CIP and AMX, and investigated for its growth-stimulating effects, antibiotic removal capabilities, and its effects on the biochemical composition of algal cells compared to the control medium for 7 days. The results demonstrated that *C. vulgaris* adapted the antibiotic spiked medium and removed CIP (37 ± 2%) and AMX (25 ± 3%), respectively. The operating mechanisms were bioadsorption, followed by bioaccumulation, and biodegradation, with an increase in cell density up to 46 ± 3% (CIP) and 36 ± 4% (AMX), compared to the control medium. Further investigations revealed that, in the CIP stress-induced algal medium, an increase in major photosynthetic pigment chlorophyll-a (30%) and biochemical composition (lipids (50%), carbohydrates (32%), and proteins (65%)) was observed, respectively, compared to the control medium. In the AMX stress-induced algal medium, increases in chlorophyll-a (22%), lipids (46%), carbohydrates (45%), and proteins (49%) production were observed compared to the control medium. Comparing the two different stress conditions and considering that CIP is more toxic than AMX, this study provided insights on the photosynthetic activity and biochemical composition of *C. vulgaris* during the stress conditions and the response of algae towards the specific antibiotic stress. The current study confirmed the ability of *C. vulgaris* to adapt, bioadsorb, bioaccumulate, and biodegrade emerging contaminants. Moreover, the results showed that *C. vulgaris* is not only able to remove CIP and AMX from the medium but also can increase the production of valuable biomass usable in the production of various bioproducts.

## 1. Introduction

Antibiotics are used in veterinary, human, and aquaculture targeting relative to the bacterial community to prevent or treat microbial diseases and infections. These antibiotics reach the aquatic environment persistently through various routes such as veterinary and human excretions, hospital wastewater, pharmaceutical wastewater, and sewage, reaching treatment plants in concentrations ranging from nanogram per liter to milligram per liter [1]. When properly untreated, the antibiotics reach the environment, causing chronic toxicity to some non-target organisms as they are designed to induce a biological response in living organisms [2]. The major problem associated with antibiotic polluted water is the development of antibiotic-resistant bacteria (ARB) and antibiotic-resistant genes (ARGs), which are responsible for 700,000 deaths per year. The treatment of ARB is highly problematic as they have developed resistance towards the antibiotics that are particularly prescribed for the treatment [3]. Ciprofloxacin (CIP) is an important antibiotic of the class of fluoroquinolones that targets Gram-positive and Gram-negative bacteria to treat severe infections, and its emission is remarkably found in municipal wastewater (58% of its total amount) and surface water (25% of its total emission) globally [4]. Amoxicillin (AMX) belongs to the β-lactam group, which accounts for more than 65% of the global antibiotic market and is recognized as one of the major threats to the environment as it is being frequently detected in surface waters [5,6]. The maximum detected worldwide concentration of CIP and AMX is 6.5 mg/L and 1.67 µg/L, respectively, with a median concentration range of 10–100 ng/L [7,8,9]. Their presence in the ecosystem is known to have effects on microorganism communities, such as bacteria, algae, invertebrates, and crustaceans. The effects of antibiotics in the aquatic ecosystem depend upon acute aquatic toxicity EC_50_ (concentration of pollutant that inhibits 50% of growth) present in water streams as they can inhibit the growth of beneficial microorganisms such as prokaryotes and eukaryotes [10].

Microalgae are eukaryotic organisms that play a crucial role in the production of oxygen in the aquatic ecosystem, as well as an important part of the food chain. Microalgae have drawn attention in the bioremediation research community for being non-target organisms for antibiotics and their ability to adapt and remove the antibiotics themselves from polluted water, producing valuable biomass [11,12,13]. *Chlorella vulgaris,* a unicellular microalga, is one of the most commonly investigated algae in the treatment of wastewater as it is commonly found in freshwater and soil habitats with fast growth rates and short production time when employed [14]. Antibiotic presence in the wastewater promotes dual responses on *C. vulgaris,* which either includes inhibitory effects or growth stimulation effects based upon the concentration [15]. Inhibitory effects depend on the EC_50_ value after 96 h of exposure. It has been reported that the 96 h EC_50_ value of *C. vulgaris* for CIP is 20.6 mg/L and 96 h EC_50_ for *P. subcapitata* when AMX is less than 50 mg/L [16,17]. *C. vulgaris* is known to adapt to antibiotics stress conditions by spontaneous physiological adaptation and can biodegrade pollutants, becoming a suitable candidate for antibiotic removal from wastewater [18]. Under stress and nutrient limitation, *C. vulgaris* is known to accumulate lipids and store energy in the form of proteins and carbohydrates.

In this study, the growth-stimulation effect caused by the presence of two different antibiotic stress conditions was investigated in comparison with the control medium. *C. vulgaris* medium was spiked with a tolerant concentration of antibiotics, considering EC_50_ value, world median concentration, maximum detected worldwide concentration, and instrument sensitivity for CIP and AMX detections during the analysis. The study lasted 7 days to investigate the mechanisms adapted by algae for the removal of CIP and AMX. The effects of these antibiotics on cell growth, photosynthetic activity (chlorophyll-a), and biochemical composition (lipids, carbohydrates, and proteins concentration) were monitored. The results were encouraging, even if the study needs further experimental runs to define the optimal operative conditions for a sound scale-up.

## 2. Materials and Methods

### 2.1. Reagents and Materials

Ciprofloxacin (CIP) (CAS No.: 85721-33-1) and amoxicillin (AMX) (CAS No.: 61336-70-7) (HPLC grade, >98% purity) stock solutions of 100 mg/L were prepared by dissolving reference standards in ultra-pure water and stored in a dark airtight Schott glass bottle at 4 °C for no longer than 10 days. The required concentration was prepared from a stock solution using serial dilution. All reagents and chemicals were of analytical grade.

### 2.2. Algae and Incubation Conditions

*C. vulgaris* (BDU GD003) was purchased from NRMC-F (National Repository for Microalgae and Cyanobacteria-Freshwater), Bharathidasan University, Tiruchirappalli, Tamil Nadu, India. The culture was maintained and sub-cultured in BG-11 medium at an orbital shaking incubator under a fluorescent light intensity of 50 µmol photon/m^2^/s in 12 h light/12 h dark cycle at 30 °C until the stationary phase was attained.

### 2.3. Experimental Setup

All the experiments were conducted in batch culture using autoclaved 250 mL Erlenmeyer flasks containing 100 mL of BG-11 medium. A series of runs was conducted for each antibiotic, namely Batch 1 (CIP) and Batch 2 (AMX). In each series, four different experimental conditions (A, B, C, and D) were established for the determination of growth and removal mechanisms by *C. vulgaris.* Table 1 summarizes these conditions. Each run was performed in triplicates. In both batches, in runs A and B, algae cell pellets were inoculated with an average cell density of 5 × 10^6^ cells/mL, and their growth profiles were monitored daily to study the impact of antibiotics.

The control (B) experiments were conducted with the same illumination conditions to elucidate the possible role of abiotic conditions in the removal of antibiotics and biomass production. In both batches, A, C, and D runs were spiked with 5 mg/L of antibiotic standard solution. The experimental runs A and B were incubated at 30 ± 1 °C under a fluorescent light intensity of 50 µmol photon/m^2^/s in 12 h light/12 h dark cycles. These experimental conditions were kept for 7 days. In each run, monitoring was carried out by taking 5 mL aliquots of medium for the determination of biomass and antibiotic concentration.

### 2.4. Determination of Algal Growth and Biochemical Composition

#### 2.4.1. Algal Growth

Algal growth can be assessed by counting the algal cells using a hemocytometer, optical density (OD), and measuring chlorophyll-a content. The number of cells per mL of *C. vulgaris* was measured using a Neubauer improved hemocytometer under 40× magnification in a trinocular microscope, and the specific growth rate (µ) was calculated by using the following equation [19]:

μ = (ln N_2_ − ln N_0_)/(t_2_ − t_0_) (1)

where N_0_ is the cell density at time t_0_ (day 0), and N_2_ is the cell density at time t_2_ (day 7).

The maximum absorbance for *C. vulgaris* was inspected by scanning sample cultures between 500 and 800 nm using a UV-Vis spectrophotometer, and the maximum absorbance was found at 680 nm [20]. On this basis, the spectrophotometer was set to 680 nm wavelength to measure the OD values, as this parameter reflects the cell density in the medium by taking refraction into account.

Chlorophyll-a extraction and the concentration of the extract were calculated using the following equation [19]:

Chlorophyll-a (mg/L) = (9.90 × OD_660_) − (0.77 × OD_642_) (2)

where OD_660_ and OD_642_ are the optical densities of the extracted chlorophyll pigment from the culture at 660 nm and 642 nm.

#### 2.4.2. Biochemical Composition

Algae cells were harvested by centrifugation for the analysis of the biochemical composition. Harvested algae pellets were subjected to sonication for the extraction of lipids and quantified gravimetrically, as reported in [21,22]. Carbohydrates were extracted using the Anthrone method and quantified (in milligram per liter) by UV-Vis spectrophotometer using glucose standards [21]. Proteins were extracted using the Lowry method and quantified by a UV-Vis spectrophotometer using bovine serum albumin (BSA) standards [21]. Dry biomass was calculated gravimetrically by drying the harvested algae cell pellets.

Lipids, carbohydrates, and proteins concentrations were calculated as percentages by biomass weight by the following equation, respectively.

%Lipids = Lipid mass/Biomass weight × 100(3)

%Carbohydrates = Carbohydrate mass/Biomass weight × 100(4)

%Proteins = Protein mass/Biomass weight × 100(5)

### 2.5. Determination of Antibiotic Concentration

CIP and AMX concentrations were determined according to the USP 28-NF 23 s supplement [23,24], using 844 UV/VIS compact ion chromatography equipped with a Hichrom HPLC column (Alltima 5 µm C18 with dimensions 250 × 4.6 mm). The mobile phase consisted of 15% acetonitrile and 85% ultra-pure water (pH = 3.0) with a flow rate of 1 mL/min and 250 µL injection volume. The wavelength of the UV detector for CIP and AMX was 270 nm (retention time = 25.88 min) and 230 nm (retention time = 4.15 min), respectively. All samples were filtered through a 0.22 µm membrane filter before analysis. The concentration was estimated using IC net 2.3 software integrated with ion chromatography equipment. The overlay curves of the individual sample analysis were plotted using IC net software upon the completion of all analysis.

### 2.6. Determination of Antibiotic Removal Mechanisms in Algal Cells

Aliquots of 5 mL of microalgal suspension were withdrawn and separated by centrifugation at 2500 rpm for 10 min. The filtered supernatant was then used for analyzing the residual concentration (Cr) of antibiotics in the algal medium.

As a first information, total antibiotic removal can be calculated as follows:

(6) $$\mathrm{Total}\;\mathrm{removal}\;\left(\%\right)=\frac{\mathrm{Ci}\;-\;\mathrm{Cr}\;}{\mathrm{Ci}\;}\times \;100$$

where Ci is the initial antibiotic concentration in the medium, and Cr is the residual concentration.

Antibiotics tend to be adsorbed on algal cell walls due to the interaction between the pollutant and extra polymeric substances of microalgae. This amount of antibiotic can be desorbed from the cell wall by resuspending the harvested algal pellets with 5 mL of ultra-pure water and centrifuged again carefully by increasing the rotation speed from 2500 to 5000 rpm for 10 min without disrupting the cell wall [25,26]. The filtered supernatant was used for the determination of antibiotics adsorbed on the cell wall (Rad). The bioaccumulation (Rac) mechanism was determined by using the sonication method [27]. The centrifuged pellet was again suspended by adding 5 mL dichloromethane-methanol solution (concentration 1:2 by volume), sonicated for 30 min, and then centrifuged again for the analysis of antibiotics accumulated inside the algal cells.

As aforesaid, an amount of antibiotic can be removed in the abiotic condition (R_a_) by photodegradation.

(7) $$R_{a}\;\left(\%\right)=\frac{\mathrm{Cr}\left(\mathrm{abiotic}\;\mathrm{dark}\right)\;-\;\mathrm{Cr}\left(\mathrm{abiotic}\;\mathrm{light}\right)\;}{\mathrm{Ci}\;}\;\times \;100$$

At last, the biotic removal (R_b_) of antibiotics in the *C. vulgaris* medium was calculated by the equation given by Xiong et al. [28], taking into account the adsorption, bioaccumulation, and abiotic removal.

(8) $$R_{b}\;\left(\%\right)=\frac{\left(\mathrm{Ci}\;-\;\mathrm{Cr}\;-\;\mathrm{Rad}\;-\;\mathrm{Rac}\;-{\;R}_{a}\right)\;}{\mathrm{Ci}\;}\;\times \;100$$

### 2.7. Statistical Analysis

All experiments were carried out in triplicates and the average results were reported. The data obtained from different experimental conditions were compared by ANOVA, having statistical significance at *p* < 0.05. All statistical analyses and graph plotting were carried out with JMP 16.2 software.2.1.1.

## 3. Results

### 3.1. Effect of Antibiotics on Algal Growth

**Figure 1:**
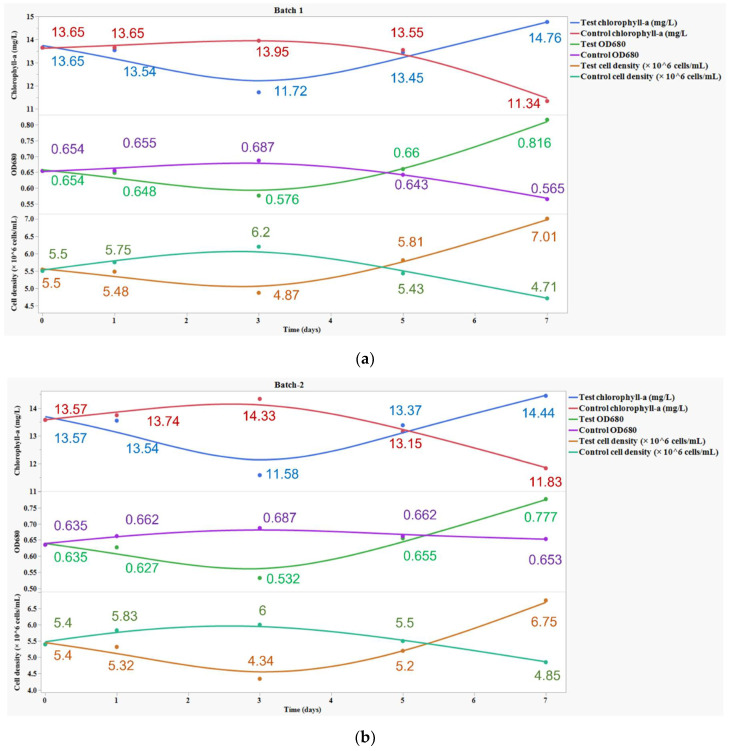
Effect of (**a**) CIP and (**b**) AMX on chlorophyll-a concentration, OD_680_, and cell density.

*C. vulgaris* growth in the experimental conditions A and B for Batch 1 and Batch 2 was evaluated, and the results are shown in Figure 1. Previous toxicological studies have shown that a low concentration of antibiotics in the system can stimulate algal growth, whereas a concentration higher than EC_50_ will have toxic effects on the algal structures [15,29,30].

Figure 1a,b show the effect of CIP (a) and AMX (b), respectively, on the chlorophyll-a concentration, OD_680_, and cell density in runs A and B for the tested batches.

In both batches, these parameters declined until day 3 in experimental condition A, compared to run B, indicating the stress caused by antibiotics upon the culture in the medium.

On day 5, in both batches, there was an increase in chlorophyll-a concentration, OD_680_, and cell density for run A, indicating that the response mechanisms of *C. vulgaris* adapted and grew in the stress conditions induced by CIP and AMX.

The specific growth rate for the experimental condition B in Batch 1 was found to be −0.03 d^−1^ and −0.029 d^−1^ for Batch 2. A negative growth rate indicates the decline of algal cells due to the absence of nutrients in the medium, whereas for run A, it was found to be 0.04 d^−1^ in Batch 1 and 0.038 d^−1^ in Batch 2, respectively. A positive growth rate indicates the utilization of antibiotics as a carbon source for their growth.

To summarize, for both batches, the results revealed that induced stress caused by CIP and AMX stimulated algal growth. This is clearly evidenced by the comparison of the data achieved in runs A and B.

### 3.2. Effect of Antibiotics on Algal Biochemical Composition (Lipids, Carbohydrates, and Proteins)

Cell growth and biochemical composition accumulation are closely related to the adopted environmental conditions, such as light intensity, nutrient concentration in the medium, stress conditions, and CO_2_ concentration [31,32,33]. During algal photosynthesis, chlorophyll transforms light energy into adenosine triphosphate (ATP) and nicotinamide adenine dinucleotide phosphate (NADPH), which converts the carbon source and CO_2_ into glyceraldehydes-3-phosphate (G3P) during reaction in dark conditions [34]. G3P in the glycolytic pathway results in the biosynthesis of carbohydrates, while a part of G3P will be transformed into acetyl-CoA and pyruvate by the action of glycolysis and take part in the formation of protein. Acetyl-CoA catalyzed by acetyl-CoA carboxylase is converted into malonyl-CoA, which plays an important role in the synthesis of fatty acids. The produced fatty acids becomes accumulated in the form of lipids in the chloroplast of the cell [35]. Chlorophyll-a, as a major light-harvesting pigment, releases protons during photosynthesis and enhances acetyl-CoA carboxylase activities inside the cell, thus increasing the production of biochemicals inside the cell.

**Figure 2:**
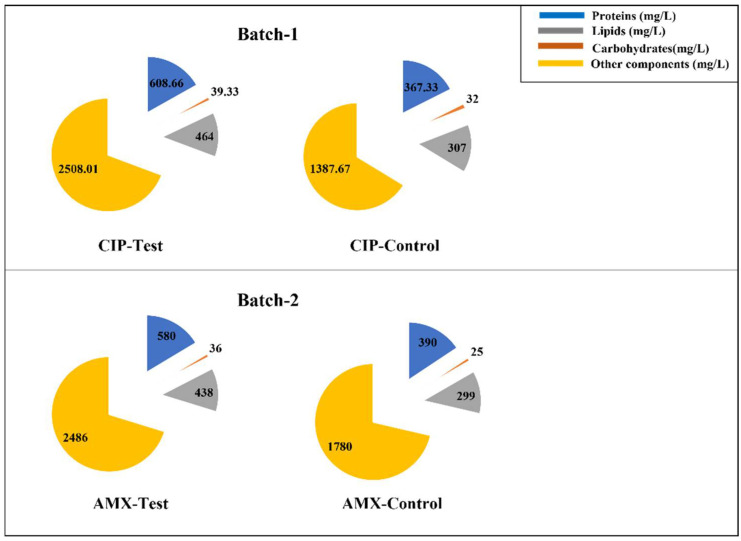
Effect of CIP and AMX upon lipid, carbohydrate, and protein accumulation in *Chlorella vulgaris* (incubation time = 7 days).

Figure 2 reports the results achieved in the runs. The data refer to the lipid, carbohydrate, and protein concentration at the end of runs A and B after 7 days of incubation.

In this study, it was observed that lipids, carbohydrates, and protein increased in both the tested batches (1 and 2) compared to the control conditions, as shown in Figure 2, and the biomass dry weight for the experimental condition A of Batch 1 and Batch 2 is 72% and 41% higher than the control conditions. Furthermore, it can be noted that in run A of Batch 1, the lipid (464 mg/L), carbohydrate (39 mg/L), and protein (608 mg/L) accumulations were higher than the ones achieved in run A of Batch 2, where lipid, carbohydrate, and protein concentrations were 438 mg/L, 36 mg/L, and 580 mg/L, respectively (Figure 2). *C. vulgaris* adapted to CIP stress conditions and utilized this antibiotic better than with AMX. Previous studies have shown that the EC_50_ value for CIP is very low compared to AMX [16,17]. This indicates that, where CIP is more toxic to algae, it induces stress on *C. vulgaris* and because of this stress, there is an increase in chlorophyll-a, lipid, carbohydrate, and protein production in run A of Batch 1 compared to experimental condition A of Batch 2.

### 3.3. Antibiotics Removal Mechanisms Adopted by Algae

Removal mechanisms were determined from the experimental conditions given in Table 1. Bioadsorption, bioaccumulation, and biodegradation are major removal mechanisms adopted by algae to remove the organic contaminants [28]. The contribution of each mechanism to the total removal is shown in Table 2.

In this study, the removal of CIP and AMX via photodegradation (abiotic condition) is determined in runs C and D. This mechanism was negligible for CIP under the tested conditions, as shown by the value reported in Table 2.

Bioadsorption is an extracellular mechanism accomplished by the polymer assemblages (cellulose, hemicellulose, and proteins) and functional groups present on the cell wall [36]. Bioadsorption and bioaccumulation were not the major removal mechanisms in this study, and even other researchers have reported that they lead to the process for the biodegradation mechanism inside the cell.

**Figure 3:**
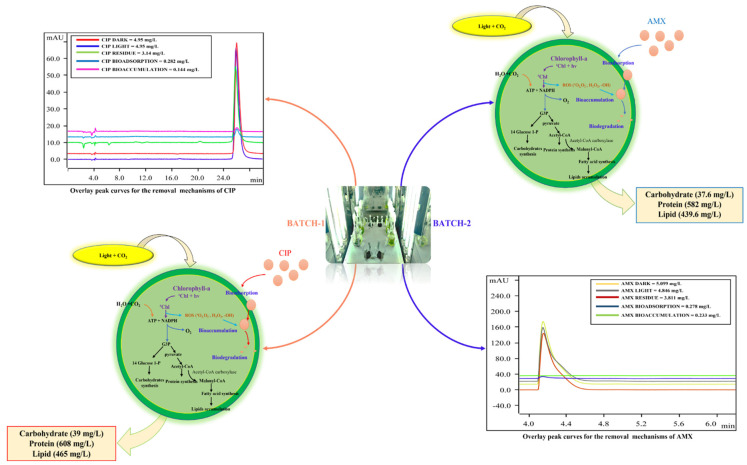
Removal mechanisms adopted by algae for their growth and their effects on lipids, carbohydrates, and proteins.

In the current study, biodegradation accounts for the major removal mechanism, 76% for CIP and 46% for AMX, respectively. These values indicate the utilization and breakdown of antibiotics as a carbon source for their cellular growth. Figure 3 shows the process involved in the removal of antibiotics by algae and its effects on biochemical composition with the overlay peak curves of HPLC analysis performed to determine the contribution of each mechanism for the removal of antibiotics.

The increase in chlorophyll-a concentration, cell density, and biochemical composition (lipids, carbohydrates, and proteins) in the test conditions (run A), compared to the control medium (run B), indicates the response mechanism adopted by algae to utilize CIP and AMX after the depletion of nutrients in the medium.

## 4. Discussion

Studies have reported that algae produce free radicals such as peroxyl radicals, single oxygen, and hydroxyl radicals during photolysis [37]. In photosynthesis, a single electron of chlorophyll molecule is excited to a higher energy state within the photosystem to form an excited triple-state chlorophyll molecule and produces free radicals in the medium. This helps to the breakdown of carbon sources for their growth [38] and at the same time for the production of lipids, carbohydrates, and proteins inside the algae themselves. However, the amounts of these biochemicals depend on the kind of algae and antibiotics.

About antibiotics, their removal depends strictly on their classes; that is to say that different antibiotics can be removed at different extents, even when they are present at the same concentrations.

The global average concentration of CIP and AMX in the surface freshwaters is in the range of 10–100 ng/L, whereas the maximum detected concentration of CIP in a lake is in the range of 2.5–6.5 mg/L [8]. In our study, we conducted the experimentation at a fixed concentration of 5 mg/L to provide insights on the impacts of these pollutants at selected concentrations. Antibiotics at lower concentrations have the potential to alter the community structure of algae in the surface waters, and it has been reported that altering the community structure can contribute to algal blooms [39]. The present study demonstrated that a concentration of 5 mg/L CIP and AMX not only stimulated growth but also removed antibiotics from the media by the mechanism of bioadsorption, bioaccumulation, and biodegradation.

The bioadsorption of compounds can be assessed by the octanol and water partition coefficient (log K_ow_) value, which determines whether the compound is hydrophobic or lipophilic in nature [40]. The higher the value, the higher the adsorption of compounds onto the surface of the microorganism. The log K_ow_ value for AMX is 0.87, which is slightly higher than the one for CIP (0.28) [41]. The results demonstrate that the bioadsorption of AMX is higher than the CIP one. Bioaccumulation and bioadsorption are a continuous process, and adsorbed antibiotics become accumulated inside the cells through cell membrane diffusion [42]. AMX is more accumulated than CIP. However, being more toxic, CIP induces stress upon algae. To counteract this accumulated toxicity, *C. vulgaris* generates free radicals inside the cell by increasing the photosynthetic activity of the cell, and this helps in the process of biodegradation.

In this study, biodegradation was the major removal mechanism, followed by bioadsorption and bioaccumulation in the CIP and AMX test.

In line with this, CIP removal was higher than the AMX one, namely about 37% (CIP) against 25% (AMX). Taking these results into consideration, it is evident that *C. vulgaris* is more suitable to remove CIP than AMX. This can be justified by the chemistry of the tested antibiotics: CIP belongs to the fluoroquinolones, whereas AMX belongs to β-lactams, and their affinity to *C. vulgaris* is different.

It is interesting to note that, for both antibiotics, the chlorophyll-a concentration, cell density, and specific growth rates are very similar after 7 days.

About CIP, the achieved removal efficiency is lower than the one obtained by Hom-Diaz et al. [30], who studied the removal of this antibiotic by *C. sorokiniana*. Its efficiency was around 50% after 14 days, starting with a CIP concentration equal to 0.1 mg/L, which is much lower than the value used in the current study (5 mg/L). The longer process duration (more than double) could justify the higher removal efficiency, more than the different genus of *Chlorella*. Hom-Diaz et al. [30] found photodegradation as the main mechanism responsible for CIP removal. This result is in contrast with what was obtained in the current study, where photodegradation does not seem to be active in CIP removal (Table 2). The use of *C. vulgaris* instead of *C. sorokiniana* could be the reason. Moreover, the photodegradation process mainly depends on light intensity. Biodegradation showed to be the main removal mechanism also in the study of Xie et al. [43], where wastewater containing 5-mg/L of CIP was treated with *Chlamydomonas* sp. Tai-03 for 9 days.

Xiong et al. [28] used *C. vulgaris* to remove levofloxacin, a fluoroquinolone, from an initial concentration equal to 5 mg/L. After 7 days, about 15% of the antibiotic was removed, which is much lower than the amount achieved in the present study (37%). However, it must be evidenced also that these authors found biodegradation as the main mechanism able to remove the antibiotic and not activity by photodegradation, as was observed in this study. The reason could be the same algal kind.

For AMX, removal efficiency was 25%. Zhao et al. [44] studied the removal of AMX by *Chlorella regularis*. In their test, AMX was initially present at a concentration equal to 3 mg/L, and after 7 days, the concentration was reduced to 45%. Their cell density was 15 × 10^6^ cells/mL, against about 7 × 10^6^ cells/mL of the current study. Zhao et al. [44] also checked the concentration of lipids, carbohydrates, and proteins after 18 days, achieving values around 420 mg/L, 120 mg/L, and 120 mg/L, respectively. Notwithstanding the different removal efficiency, the lipid concentration is completely in line with the current one (440 mg/L), whereas carbohydrate concentration is higher (38 mg/L, in the current study), and the protein concentration is much lower (in this study, it is 580 mg/L). The comparison of these concentrations is not so easy due to the different processing times, which for Zhao et al. [44] was three times more. 

A similar study was carried out by Zhang et al. [33] for the removal of AMX (starting concentration = 5 mg/L) by *C. regularis* for 5 days. At the run’s end, about 90% of AMX was still present in the medium. At that time, cell density was around 3.5 × 10^6^ cells/mL, and this value can confirm the low removal efficiency. The authors also analyzed the concentration of lipids, carbohydrates, and proteins at day 5, and the results were rather low at about 95 mg/L, 13 mg/L, and 13 mg/L, respectively. These values are much lower than the concentrations achieved in the current study. Both studies making use of *C. regularis* show that the concentrations of lipids are always much higher than the other compounds (carbohydrates and proteins), which have the same concentration, even if at a different values: 120 mg/L in the study of Zhao et al. [44] and 13 mg/L in the study of Zhang et al. [33]. This does not hold for the current study, where protein production reached a very high concentration and was always over the lipid’s concentration. At the moment, no hypothesis can be suggested and verified for this.

The need for further studies is evident to optimize the process in view of its scale-up.

This study provides new insights in utilizing microalgae for the treatment of CIP and AMX-polluted wastewater to remove these antibiotics. Moreover, the results demonstrated a second advantage linked to the removal and represented by valuable biomass production, containing lipids, carbohydrates, and proteins.

## Figures and Tables

**Table 1:** Experimental setup conditions and their respective abbreviations.

Batch 1—CIP	Experimental Condition	Culture	Antibiotic	Illumination
A	CIP Test	Algae	+	+
B	CIP Control	Algae	-	+
C	CIP Abiotic light	-	+	+
D	CIP Abiotic Dark	-	+	-
**Batch 2—AMX**	**Experimental Condition**	**Culture**	**Antibiotic**	**Illumination**
A	AMX Test	Algae	+	+
B	AMX Control	Algae	-	+
C	AMX Abiotic light	-	+	+
D	AMX Abiotic Dark	-	+	-

**Table 2:** Antibiotic removal (%) and removal contribution (%) of mechanisms after 7 days.

Antibiotic	Total Removal	Photodegradation	Bioadsorption	Bioaccumulation	Biodegradation
CIP	36.9 ± 1.1	0.54 ± 0.02	15.4 ± 0.4	7.92 ± 0.06	76.09 ± 0.55
AMX	24. 7 ± 1.0	24.44 ± 9.71	18.48 ± 5.64	10.84 ± 9.56	46.23 ± 5.89
